# Maternal obesity remodels nutrient transport transcriptional programs in early mouse embryonic and extraembryonic cell lineages

**DOI:** 10.1016/j.molmet.2026.102375

**Published:** 2026-04-30

**Authors:** Amalia Caballero, Lijun Chi, Paul Delgado-Olguín

**Affiliations:** 1 Translational Medicine, The Hospital for Sick Children, Toronto, ON, M5G0A4, Canada; 2 Department of Molecular Genetics, University of Toronto, Toronto, ON, M5S1A8, Canada; 3 Heart and Stroke Richard Lewar Centre of Excellence in Cardiovascular Research, Toronto, ON, M5S3H2, Canada

**Keywords:** Maternal obesity, Disease programming, snRNA-seq, snATAC-seq, Retinol transport, Lipoprotein transport

## Abstract

**Background:**

Maternal obesity increases the risk of congenital anomalies and later-life metabolic disease in offspring. Still, underlying mechanisms remain unclear, particularly in extraembryonic lineages at the maternal–fetal interface, which remain poorly studied.

**Methods:**

We jointly profiled gene expression and chromatin accessibility in single nuclei from mouse embryos and extraembryonic tissues in a diet-induced obesity model at embryonic day 8.5, when multiple organogenesis programs are underway.

**Results:**

This analysis generated an atlas of 36 cell lineages, including derivatives of all three germ layers and trophoblast populations. Lineage allocation was preserved in embryos from obese dams. However, transcription was widely dysregulated. Oxidative phosphorylation genes were broadly suppressed, and genes involved in hypoxia, cytoskeleton remodeling, and cell migration were enriched among upregulated pathways. Chromatin accessibility changed in a few lineages, most notably in extraembryonic visceral endoderm and parietal trophoblast giant cells. Differently accessible chromatin was enriched in binding motifs for retinoic acid receptors. Indeed, genes involved in retinol and lipoprotein transport were suppressed, and RNA *in situ* hybridization confirmed reduced expression of retinol transporters *Ttr*, *Rbp4*, and *Stra6,* and lipoprotein transporter *Apoa1* in visceral yolk sac.

**Conclusion:**

Obesity during pregnancy causes early transcriptional dysregulation that impairs retinoic acid and lipoprotein transport at the maternal–fetal interface, suggesting a mechanism through which maternal obesity could influence long-term developmental outcomes.

## Highlights

• Single-nucleus Multiome maps embryonic and extraembryonic responses to obesity.
• Maternal obesity broadly alters transcription in mouse embryos at organogenesis onset.
• Extraembryonic lineages show the strongest gene expression and chromatin changes.
• Maternal-fetal retinol and lipoprotein transport programs are downregulated.
• Impaired maternal–fetal nutrient transport could contribute to fetal programming.

## 1 Introduction

Obesity during pregnancy is a growing public health concern. Since 1980, global rates of overweight and obesity have risen steadily in both men and women [1]. Obesity is now the most common health complication in pregnancy [2], with its prevalence increasing from 4.7% before 1990 to 16.3% between 2010 and 2019 [3]. As this trend continues, more pregnancies will be affected. Understanding the consequences of obesity during pregnancy is therefore critical for improving pregnancy outcomes and protecting offspring's health.

Epidemiological and experimental studies show that excessive gestational weight gain and pre-pregnancy obesity increase the risk of adverse developmental outcomes in offspring [4]. Obesity during pregnancy programs long-term metabolic dysfunction [5], increasing the risk of obesity, type 2 diabetes [6], cardiovascular disease [[7], [8], [9]], and neurodevelopmental disorders [10], among other health trajectory deviations in childhood and adulthood. The developmental origins of health and disease (DOHaD) framework proposes that the intrauterine environment influences organ development and thus lifelong health trajectories [11]. Maternal obesity is associated with increased incidence of congenital anomalies, including congenital heart disease, neural tube defects and urogenital malformations [[12], [13], [14]]. This suggests that developing progenitor cells are highly sensitive to maternal metabolic status during organogenesis, and that the long-term effects of exposure to obesity *in utero* could originate as disruption of gene regulatory processes during early embryogenesis. For example, cardiac progenitor cells isolated from mouse embryos of obese dams have dysregulated metabolic and developmental gene networks linked to adult-onset heart disease susceptibility [15]. In adulthood, these offspring develop mild heart dysfunction and respond with exacerbated myocardial remodeling to cardiovascular stress [15]. This suggests that an *in utero* obesogenic environment alters transcriptional regulation in the developing heart, potentially leading to long-term negative effects in adult life. Moreover, single-cell RNA sequencing (scRNAseq) studies have revealed that maternal obesity induces transcriptional dysregulation in specific developing cell lineages. Such as increased pro-inflammatory and fibrosis responses in E13.5 mesenchymal cells and fibro-adipogenic progenitors [16]; and suppressed myogenic differentiation pathways across E9.5 myogenic progenitor lineages [17]. However, studies investigating fetal transcriptional dysregulation due to maternal obesity have largely focused on selected tissues or cell lineages, and genome-wide analyses linking transcriptional changes to chromatin accessibility during early embryonic development remain limited.

At embryonic day 8.5 (E8.5), lineages from all three germ layers are proliferating, and multiple progenitor lineages are being specified [[18], [19], [20]]. At this stage, the placental vasculature is not functional yet [21] and extraembryonic lineages, including the visceral yolk sac and trophoblast, mediate maternal–fetal nutrient uptake, processing, and delivery to the embryo proper [[22], [23], [24]]. This interface is also essential for the transfer of key micronutrients that act as developmental signals. For example, retinol transport to the embryo requires retinol-binding protein 4 (RBP4), its carrier protein transthyretin (TTR), and the retinol receptor stimulated by retinoic acid 6 (STRA6) in the extraembryonic visceral endoderm [[25], [26], [27]]. Obesity in pregnancy has been linked to altered circulating lipids, adipokines, inflammatory mediators, and oxidative stress [[28], [29], [30]]. These changes could affect nutrient delivery to the embryo, leading to alterations to fetal growth and development. Accordingly, maternal obesity induces dysregulated placental nutrient transporter function and lipid metabolism [[31], [32], [33]]. However, the basis of nutrient transport dysregulation in developing cell lineages and the maternal–fetal interface during early organogenesis in obese pregnancies is poorly understood.

Here, we analyzed transcription and chromatin accessibility across E8.5 mouse embryonic and extraembryonic lineages in diet-induced obese and control dams by single-nucleus Multiome sequencing. This approach identified global and lineage-specific gene expression responses to maternal obesity, providing mechanistic insight into how altered nutrient handling and progenitor dysregulation could influence long-term developmental outcomes.

## 2 Methods

### 2.1 Mice

All animal procedures were approved by the Animal Care Committee at The Centre for Phenogenomics and complied with the NIH Guide for the Care and Use of Laboratory Animals. All experiments were conducted on C57BL/6J mice. Mice were housed in standard vented cages in temperature- and humidity-controlled rooms under a 12-hour light–dark cycle (21–22 °C, 30–60% humidity), with free access to food and water. From 3 weeks of age and throughout life, female mice were fed irradiated diets *ad libitum*, consisting of either a control (CT) diet (10% fat and 1% sucrose kcal; D12450K) or a high-fat/high-sugar (HF) diet (60% fat and 10% sucrose kcal; D12492i), both obtained from Research Diets Inc. Females were mated at 15 weeks of age, and only their first litters were used for experiments. All males were fed control diet except during breeding. For embryo studies, vaginal plugs were counted as embryonic day 0.5 (E0.5), and somite number was used to determine embryonic stage, with 8–12 somites indicating E8.5.

### 2.2 Weight tracking and body composition analysis

Body weight of female mice was measured weekly beginning at 3 weeks of age, the onset of diet feeding. Body composition of un-anesthetized 13-week-old female mice was measured using the body composition analyzer (EchoMRI-100 machine, Echo Medical Systems, Houston, TX, USA). Fat percentage was calculated by dividing fat mass by body weight. Lean percentage was calculated by dividing lean mass by body weight. Body weight used for these calculations was taken immediately before the placement of mice in the body composition analyzer.

### 2.3 Metabolic cage analysis

14-week-old CT and HF female mice were placed in the Promethion metabolic cage system (Sable Systems, Las Vegas, NV). The Promethion metabolic cage is an open-circuit indirect calorimetry system that records energy expenditure (EE) and mouse activity levels every 30 min for as long as mice are placed in the cages. EE is a measurement of energy consumed to sustain vital functions such as respiration, circulation, and digestion. It was calculated from the ratio of carbon dioxide (CO2) produced and oxygen (O2) consumed as a result of substrate food oxidation. CT and HF mice were kept in the Promethion temperature- and humidity-controlled cages for 4 days with 12-hour light–dark cycles (21–22 °C, 30–60% humidity), and free access to their respective diet and water. The resulting data was analyzed using the CalR software [34]. Calorie consumption was calculated by multiplying the difference in weight of the food, before and after placing mice in the cages, by the calories per gram of each diet (CT diet = 3.82 kcal/g, HF diet = 5.21 kcal/g). The diet calorie content was obtained from the manufacturer.

### 2.4 Histology

Freshly dissected perigonadal white adipose tissue (WAT), supraspinal brown adipose tissue (BAT), and liver were stored in 4% paraformaldehyde (4% PFA) overnight and prepared for paraffin embedding following previously published protocols [35,36]. All tissues were sectioned at a thickness of 7 μm and stained with hematoxylin and eosin (H & E). The white and brown adipocyte cross-sectional area was measured on 60 randomly chosen adipocytes per sample using ImageJ.

### 2.5 RNA extraction

BAT and liver tissue were collected and frozen in 300 μl of Trizol reagent (Thermo Fisher Scientific). Tissues were homogenized using the TissueLyserII (Qiagen) at 250 kHz for 2 min. Homogenized samples were centrifuged to collect supernatant. RNA isolation was performed using the Direct-zol™ RNA MiniPrep kit (Zymo Research).

### 2.6 Quantitative reverse transcription PCR

cDNA was prepared using 500 ng of extracted RNA using the Wisent cDNA synthesis kit (Wisent INC). PCR reactions were prepared using advanced qPCR MasterMix (Wisent INC). All samples were run in triplicate. Data were analyzed using the CFX Manager Software (Bio-Rad). The following primers were used: tumor necrosis factor α (*Tnfα*) 5′-3′(CTTCTGTCTACTGAACTTCGGG), 3′-5′(CAGGCTTGTCACTCGAATTTTG); Interleukin 1 beta (*Il1b*) 5′-3′(ACGGACCCCAAAAGATGAAG), 3′-5′(TTCTCCACAGCCACAATGAG); Monocyte marker monocyte chemoattractant protein 1 (*MCP-1*) 5′-3’ (GATGCAGTTAACGCCCCACT), 3′-5’ (CTTGAGCTTGGTGACAAAAACTACA); and Ribosomal Protein L13a (*Rpl13a*): 5′-3’ (TCCCTCCACCCTATGACAAG), 3′-5’ (GTCACTGCCTGGTACTTCC).

### 2.7 Glucose tolerance test

Glucose tolerance tests were performed on 15-week-old female mice after 16 h of fasting with *ad libitum* access to water. Blood glucose was measured after the fasting period (0 min) to detect fasting blood glucose levels. To analyze glucose tolerance, mice received an intraperitoneal injection of glucose (1 mg/g of body weight), and blood glucose levels were measured 15 min, 30 min, 60 min, and 120 min post injection. Blood glucose was measured using a glucometer (Contour NEXT, Bayer HealthCare).

### 2.8 Insulin tolerance test

Insulin tolerance tests were performed on 15-week-old female mice after 6 h of fasting with *ad libitum* access to water. Blood glucose was measured after the fasting period (0 min) to detect fasting blood glucose levels. To analyze insulin tolerance, mice received an intraperitoneal injection of insulin (0.6 U/Kg of body weight), and blood glucose levels were measured 15 min, 30 min, 60 min, and 120 min post-injection. Blood glucose was measured using a glucometer (Contour NEXT, Bayer HealthCare).

### 2.9 Embryo collection

Uteri were collected in ice-cold 1X PBS. Embryos were immediately dissected, and E8.5 embryos with 8–12 somites were placed in cryogenic storage vials. The vials were snap-frozen in liquid nitrogen and stored in liquid nitrogen tanks. For each sample, 4–5 embryos from the same developmental stage and the same litter were pooled. Only one pooled sample was generated per litter. Three independent litters from CT dams and three independent litters from HF dams were collected for single-nuclei analysis. Nuclei isolation and 10x Genomics Single Cell Multiome ATAC + Gene Expression v1 sequencing were performed by the Princess Margaret Genomics Centre (PMGC).

### 2.10 Single-nuclei RNA and ATAC sequencing

snRNA-seq and snATAC-seq files were individually processed with 10x Genomics Cell Ranger V2.0 using default parameters. Genome annotation: Gene reads were mapped to the *Mus musculus* mm10 genome (Ensembl genome annotated assembly GRCh38.98). Peak quantification: First, MACS2 V2.1.9.1 was used to quantify ATAC peaks for each sample. Second, to ensure all peaks were aligned between samples, a unified set of peaks was generated using the reduce function from GenomicRanges V1.54.1. Finally, peaks in nonstandard chromosomes and blacklisted mm10 genomic regions from the ENCODE Project Consortium were discarded [37]. The resulting fragment files were used to re-quantify peaks using Seurat V5.0.1. Quality control: Individual cell libraries with fewer than 1000 RNA and ATAC counts were excluded. Cell libraries with low expression complexities of less than 1000 genes were excluded. Cells with mitochondrial expression fractions larger than 40% were excluded. Doublet removal: First, an RNA-based doublet score was calculated for each sample using the runscrublet function from SinglecellTK V1.19.1 R package for each 10x sample. Cells that had scrublet scores higher than 0.2 were classified as doublets and removed. The Propeller function in Speckle V1.10.0 was used to determine whether each cell cluster was present in similar proportions across diet conditions and samples. Samples were merged after quality control filtering. Normalization: 39,802 nuclei were retained for subsequent analysis. Log-normalization, highly variable gene (HVG) selection, data scaling (with mitochondrial transcript fraction, ribosomal transcript fraction, and cell cycle stage set as vars.to.regress), and dimensionality reduction were performed using Seurat V5.0.1 on the snRNA-seq data. Term frequency-inverse document frequency (TF-IDF) normalization, feature selection, and singular value decomposition (SVD) were performed using Signac V1.12 on the snATAC-seq data. Clustering: Seurat's FindMultiModalNeighbors and FindClusters with default options were used to generate a weighted nearest neighbor (WNN) graph using Principal components (PC) 1–50 from the snRNA-seq data and latent semantic indexing (LSI) 2–50 from the snATAC-seq data. Clusters were called from this graph using the Smart Local Moving (SLM) algorithm. Visualization: UMAP graphs were constructed for both RNAseq and ATACseq data. PC 1–50 from the snRNA-seq data and LSI 2–50 from the snATAC-seq data. Annotation: A publicly available reference E8.5 mouse atlas was used to annotate cell clusters. Seurat's reference mapping pipeline with default settings was used to project single-nuclei data onto the reference dataset, and predicted cell-cluster names were imported from the reference atlas. To confirm that the annotations were correct, cell-type-specific gene expression was examined for each cluster. For cell clusters that did not highly express a known cell-type marker, the mouse Gene Expression Database (GXD) was used to assign identity based on their top 100 expressed genes.

### 2.11 Differential gene expression analysis

Seurat V5.0.1 FindMarkers with default settings was used to analyze differential gene expression (DGE) between diet conditions. Ribosomal and hemoglobin transcripts were discarded for DGE analysis, as these are abundantly expressed and can obscure gene expression differences [38,39]. Bonferroni adjusted P values < 0.05 and a fold change (FC) of at least 0.1 were considered significant. Seurat V5.0.1 FindMarkers with default settings was used to analyze DGE between HF and CT cell types. Ribosomal and hemoglobin transcripts were discarded for DGE analysis. Only genes that were expressed in at least 10% of cells within each cluster were tested. Bonferroni adjusted *P*-values <0.05 were considered significant. For visualization, volcano plots were generated with EnhancedVolcano V1.2.

### 2.12 Gene Ontology enrichment analysis

EnrichR V3.4 with default settings was used to perform Gene Ontology (GO) (2023 release) on significant upregulated and downregulated genes across the entire embryo.

### 2.13 Reactome enrichment analysis

EnrichR V3.4 with default settings was used to perform Reactome enrichment analysis (2024 release) on significant upregulated and downregulated genes across the entire embryo. The same protocol was used to perform Reactome enrichment analysis on differentially accessible genes in extraembryonic visceral endoderm and parietal trophoblast giant cells.

### 2.14 Gene Set Enrichment Analysis

Gene Set Enrichment Analysis (GSEA) was performed in individual cell types using clusterProfiler V3.20 gseGO with org.Mm.eg.db V3.18 gene set database. All expressed genes within each cell type were ranked by their average log2-fold value. GSEA permutations were set at 10,000. To prevent gene sets that are too general from overtaking the results, gene sets with more than 800 genes were excluded. To prevent small gene sets from being ignored, gene sets with at least 3 genes were included in the analysis. Bonferroni adjusted *P*-values <0.05 were considered significant. For visualization, dot plots were generated using enrichplot V1.22.

### 2.15 Kyoto Encyclopedia of Genes and Genomes pathway enrichment analysis

ShinyGO V0.8 with default settings was used to perform Kyoto Encyclopedia of Genes and Genomes (KEGG) pathway enrichment analysis (release 82.0) on significantly upregulated and downregulated genes within individual cell types.

### 2.16 Differential chromatin accessibility analysis

Seurat V5.0.1 FindMarkers function was used to perform differential accessibility (DA) analysis between HF and CT cell types. Only peaks that were detected in at least 5% of cells within each cluster were tested. A logistic regression model was used to test for significance. To mitigate the effect of differential sequencing depth between cell types, the total number of snATAC-seq sequenced fragments was set as a latent variable in the logistic regression model. Bonferroni adjusted p-values <0.05 were considered significant. Signac V1.12 ClosestFeature function was used to locate the genes closest to each differentially accessible peak. Signac V1.12 LinkPeaks was used to link accessible chromatin peaks to potential gene targets based on the correlation between chromatin accessibility and gene expression. The genomic coordinates of each differentially accessible peak were searched in Ensembl's mouse genome reference GRCm38.p660 to determine the type of region each differentially accessible region falls in, *i.e*., promoter flanks, exons, introns, promoters, enhancers, transcription factor binding sites, and unknown regions.

### 2.17 Motif enrichment analysis

Signac V1.12 FindMotifs was used to find overrepresented motifs in cell lineages with more than 10 differentially accessible peaks. Briefly, this function performs a hypergeometric test for the probability of observing a TF binding motif in each peak compared to a background set of peaks with matching GC content. *Mus musculus* mm10 genome motif position frequency matrices were extracted from the JASPAR 2020 database [40] and used for motif analysis. Benjamini-Hochberg adjusted *P*-values <0.05 were considered significant.

### 2.18 ChromVar analysis

The ChromVAR V1.24 R package was used to compute per-cell TF motif activity. Briefly, this package scans every peak within individual cells for known TF motifs and calculates a z-score corrected for GC content and sequencing depth biases. This z-score measures the deviation between the observed accessibility of a given motif *versus* the expected accessibility based on the average accessibility across all cells. Larger z-scores indicate high accessibility “activity” values for a given motif. Seurat V5.0.1 FindMarkers (with mean.fxn = rowMeans and fc.name = “avg_diff”) was used to identify the top 10 active motifs within each cell lineage. Bonferroni adjusted *P*-values <0.05 were considered significant.

### 2.19 RNA *in situ* hybridization

Embryos from CT and HF females were collected as previously described in the Mice and Embryo collection sections. Freshly collected E8.5 embryos were fixed in 4% PFA for 2 h at 4 °C. Embryos were then washed in 1X PBS/DEPC for 15 min twice, and dehydrated in 10% sucrose, 20% sucrose, and 30% until they sank to the bottom. Once dehydrated, embryos were placed in OCT and stored at −80 °C. OCT blocks were cryosectioned at a thickness of 10 μm. RNAscope Multiplex Fluorescent v2 kit (ACDbio) was used to perform RNA *in situ* hybridization for the following genes: Transthyretin (*Ttr*), Retinol binding protein (*Rbp4*), Apolipoprotein a1 (*Apoa1*), Retinoic acid receptor gamma (*Rarg*), Retinoic acid receptor alpha (*Rara*), Retinoic acid receptor beta (*Rarb*), and Signaling receptor and transporter of retinol 6 (*Stra6*).

### 2.20 RNA *in situ* hybridization quantification

A Nikon A1R Confocal Microscope was used to capture fluorescence images at 10x and 60x magnification. The Qupath software was used to quantify *Ttr, Rbp4, Apoa1, Rarg, Rara, Rarb, Nr2f1*, and *Stra6* expression [41]. Briefly, this software uses DAPI staining to identify individual cells and detects subcellular structures within each cell. In the case of RNA ISH, each fluorescent spot smaller than 2 μm^2^ represents an individual mRNA transcript, while larger spots represent a cluster of mRNA transcripts.

### 2.21 Statistical analysis

Data are presented as mean ± Standard Deviation (SD) unless otherwise specified. 2 group comparisons were conducted by unpaired t-tests. For multiple group comparisons or for comparisons of 2 groups over time, ANOVA was used unless otherwise specified. Statistical analysis was conducted using GraphPad Prism 9.

## 3 Results

### 3.1 Single-nuclei transcriptomic and chromatin profiling of E8.5 embryos

To examine how obesity during pregnancy affects early mammalian development, we established a diet-induced obesity model in C57BL/6J mice that reproduced features described in previous studies, including progressive weight gain, adipocyte hypertrophy, metabolic dysregulation and inflammation [[42], [43], [44], [45]]. Female mice received either a high-fat (HF) or control (CT) diet for twelve weeks before conception. HF females progressively gained weight, had increased caloric intake, and showed reduced energy expenditure relative to activity, as quantified by the Promethion metabolic cage system (Supplementary Fig. 1). By conception, HF females had greater adiposity, enlarged white and brown adipocytes, hepatic lipid accumulation, and higher expression of inflammatory markers in brown adipose tissue and liver (Supplementary Fig. 2A–F). These changes are consistent with metabolic dysfunction often associated with diabetes risk [6,46]. However, HF females remained normoglycemic and responded to insulin similarly to CT females, although they cleared glucose more slowly (Supplementary Fig. 2G and H). Thus, prolonged HF feeding induced severe obesity without overt diabetes or hyperglycaemia, providing a model that separates the effects of maternal obesity from those of maternal diabetes on embryonic programming.

**Figure 1:**
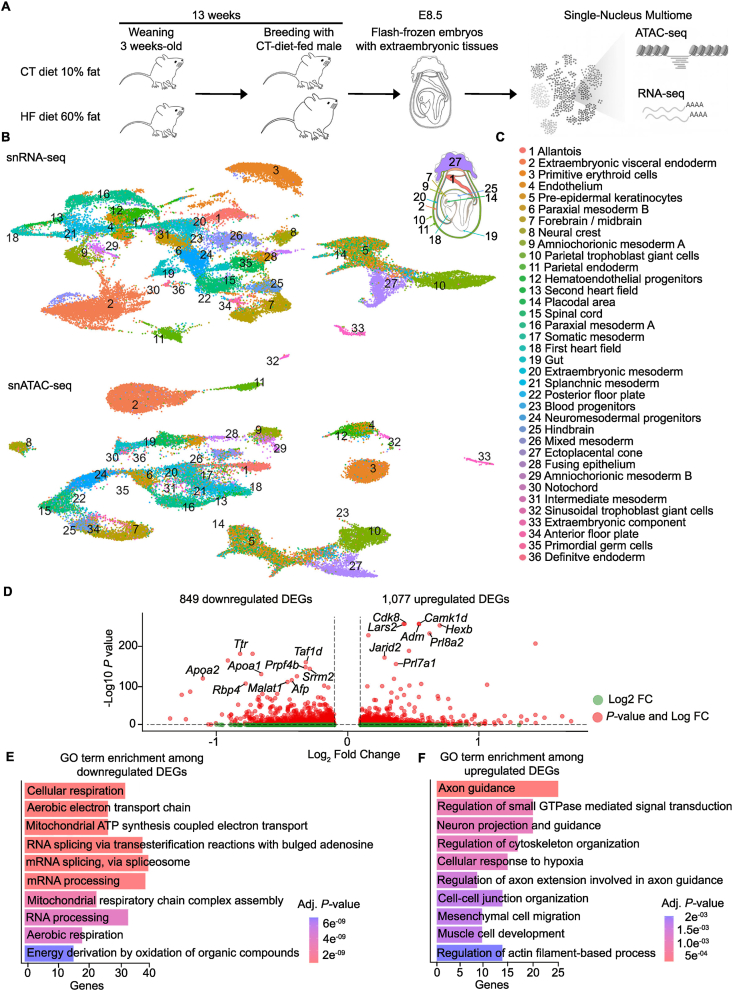
**Maternal high-fat diet alters early embryonic and extraembryonic gene expression by single-nucleus Multiome profiling. A**, Schematic of the experimental design. Female mice were weaned at 3 weeks of age and maintained for 13 weeks on either control (CT; 10% fat) or high-fat (HF; 60% fat) diets before breeding with Ctrl-diet-fed males. Embryos were collected at embryonic day 8.5 (E8.5) and flash-frozen with extraembryonic tissues. Single-nucleus Multiome was performed to jointly profile chromatin accessibility (ATAC-seq) and gene expression (RNA-seq). **B**, Uniform Manifold Approximation and Projection (UMAP) embedding of all nuclei profiled by single-nucleus RNA-seq (snRNA-seq; top) and single-nucleus ATAC-seq (snATAC-seq; bottom). Each point represents a nucleus, colored by cluster identity corresponding to distinct cell types. **C**, Annotated cell types mapped onto E8.5 embryos, illustrating the spatial organization of major embryonic lineages (numbers correspond to cluster identities in panel B). **D**, Volcano plot of genes differentially expressed in E8.5 embryos of HF mice. Red dots represent significantly dysregulated genes (adjusted *P*< 0.05 and a 0.1-fold change (FC)). Green dots represent non-significantly dysregulated genes (adjusted *P*> 0.05 0.1-FC). Dysregulated genes with the lowest adjusted *P*-values are labeled. **E**, Gene Ontology (GO) Term enrichment amongst genes downregulated in HF E8.5 embryos. Terms are coloured and organized based on adjusted *P-*values, with lower *P*-values at the top. Genes refers to the number of query genes that were detected in each GO term. **F**, GO Term enrichment amongst genes upregulated in HF E8.5 embryos. Terms are coloured and organized based on adjusted *P-*values, with lower *P-*values at the top. Genes refers to the number of query genes that were detected in each GO term.

To investigate regulatory pathways in embryonic and extraembryonic lineages, we performed simultaneous single-nucleus RNA sequencing (snRNA-seq) and ATAC sequencing (snATAC-seq) on whole E8.5 embryos attached to the yolk sac and ectoplacental cone from CT and HF dams (Figure 1A). After quality control, 39,802 nuclei passed filtering, with a median of 3,207 detected genes and 3,384 ATAC peaks per nucleus (Supplementary Fig. 3A and B). Unsupervised clustering identified 36 clusters, which we annotated using a publicly available E8.5 mouse atlas [20] and lineage-specific marker gene expression [47] (Figure 1B,C; Supplementary Fig. 3C; Supplementary Table 1). At E8.5, organogenesis is in its early stages, and cell lineages derived from ectoderm, mesoderm, endoderm, and trophectoderm expand rapidly and will contribute to extraembryonic tissues and all organs [18,48]. Accordingly, our atlas captured nine extraembryonic and twenty-seven embryonic lineages. These comprised extraembryonic visceral endoderm and trophoblast populations involved in nutrient transport before placental maturation, as well as embryonic lineages such as the first and second heart fields that contribute to distinct cardiac structures (Supplementary Table 2).

We asked whether maternal obesity alters cell expansion or differentiation at this stage. The proportions of all annotated lineages were similar in CT and HF embryos (Supplementary Fig. 3D), which suggests that maternal obesity does not overtly disrupt lineage allocation or differentiation of progenitors in E8.5 cell states. This single-nucleus Multiome atlas provides, to our knowledge, the first integrated comparison of gene expression and chromatin accessibility across embryonic and extraembryonic lineages in embryos developing under normal and obese maternal conditions.

### 3.2 Gene expression is extensively dysregulated across multiple cell lineages in embryos exposed to obesity *in utero*

To examine the global effect of maternal obesity on embryonic development, we performed differential gene expression analysis between HF and CT E8.5 embryos across all cell lineages. This analysis identified 1,926 dysregulated genes, with 849 upregulated and 1,077 downregulated (Figure 1D). Downregulated genes enriched pathways associated with cellular respiration and the electron transport chain (Figure 1E). Upregulated genes enriched gene ontologies related to axon guidance, cytoskeleton organization, mesenchymal cell migration, and cellular response to hypoxia (Figure 1F). Reactome pathway analysis showed that gene sets related to respiratory electron transport chain and aerobic respiration appear among the most enriched downregulated pathways, while Ras GTPase signaling and signal transduction appear among the most enriched upregulated pathways (Supplementary Fig. 4A and B). This suggests that maternal obesity alters embryonic transcriptional programs potentially affecting metabolic, structural, and migratory processes.

**Figure 2:**
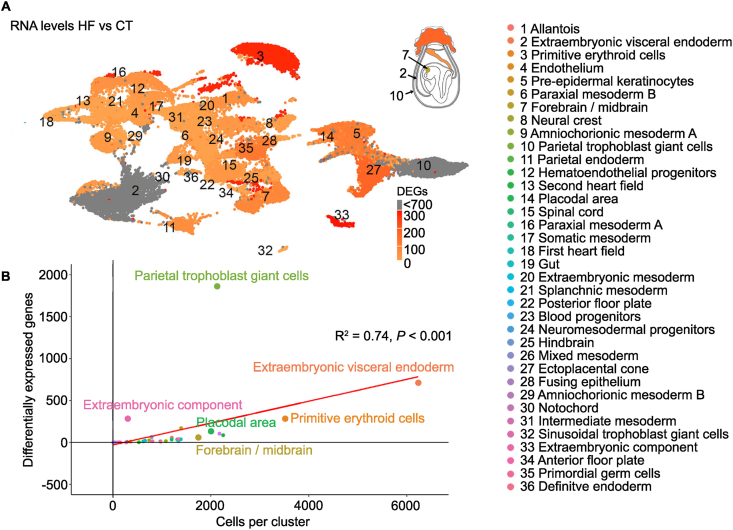
**Maternal high-fat diet alters gene expression differently across embryonic and extraembryonic cell lineages. A,** UMAP embedding of all nuclei profiled by snRNA-seq. Each point represents a nucleus, colored by the number of differentially expressed genes (DEGs). Schematic of E8.5 embryo highlights cell lineages with the highest number of DEGs (Parietal trophoblast giant cells and Extraembryonic visceral endoderm), as well as Ectoplacental cone and Forebrain/midbrain. **B**, Correlation between the number of cells (x-axis) and number of DEGs (y-axis) per cell lineage; Pearson's correlation coefficient R = 0.74, *P <*0.001. Each dot represents a cell lineage, colored by cluster identity corresponding to distinct embryonic and extraembryonic cell types (legend; right).

To define lineage-specific transcriptional responses to maternal obesity, we compared HF and CT embryos within each annotated E8.5 cell lineage. The extent of dysregulation varied across lineages. Parietal trophoblast giant cells and extraembryonic visceral endoderm showed the largest number of differentially expressed genes, with 1,863 and 713 dysregulated genes respectively (Figure 2A, Table 1). Several lineages, including definitive endoderm, intermediate mesoderm, notochord, posterior floor plate, primordial germ cells and the second heart field, showed no significant transcriptional changes.

**Table 1:** Significant differential expressed genes (DEGs) per cell lineage.

Cell lineage	˅ DEGs HF	˄ DEGs HF	Total DEGs	Total cell number
Parietal trophoblast giant cells	187	1676	1863	2139
Extraembryonic visceral endoderm	185	528	713	6233
Extraembryonic component	6	286	292	294
Primitive erythroid cells	150	126	276	3518
Ectoplacental cone	101	69	170	1390
Placodal area	92	34	126	2017
Pre-epidermal keratinocytes	50	56	106	2175
Forebrain/midbrain	47	39	86	2251
Parietal endoderm	40	26	66	790
Allantois	39	18	57	1201
Paraxial mesoderm A	32	23	55	1751
Spinal cord	19	22	41	1333
Mixed mesoderm	22	16	38	1382
Hematoendothelial progenitors	15	22	37	1312
Amniochorionic mesoderm A	22	14	36	1368
Gut	17	17	34	1196
Endothelium	14	11	25	739
Neural crest	11	10	21	652
Somatic mesoderm	3	13	16	805
Extraembryonic mesoderm	9	6	15	1079
Neuromesodermal progenitors	8	7	15	1335
Hindbrain	4	10	14	619
Paraxial mesoderm B	6	7	13	968
Splanchnic mesoderm	1	9	10	972
Amniochorionic mesoderm B	2	7	9	361
Fusing epithelium	0	6	6	286
Blood progenitors	0	4	4	284
First heart field	0	2	2	529
Sinusoidal trophoblast cells	1	1	2	132
Anterior floor plate	0	1	1	149
Definitive endoderm	0	0	0	67
Intermediate mesoderm	0	0	0	90
Notochord	0	0	0	30
Posterior floor plate	0	0	0	81
Primordial germ cells	0	0	0	6
Second heart field	0	0	0	268

We evaluated whether lineage size contributed to this variation, since larger populations in single-cell analyses provide greater statistical power [49]. Although lineage size influenced DEG detection, it did not fully account for the observed differences. Several lineages deviated from the expected relationship between cell abundance and number of dysregulated genes (Figure 2B). Parietal trophoblast giant cells produced 1,863 dysregulated genes despite having substantially fewer nuclei than extraembryonic visceral endoderm, which contained more than three times as many nuclei, yet only had 713 dysregulated genes. Forebrain/midbrain lineages contained more than two thousand nuclei but yielded only 86 dysregulated genes. Thus, obesity during pregnancy induces heterogeneous transcriptional responses across lineages. Moreover, some developing populations mount disproportionately large transcriptional changes relative to their size, suggesting increased sensitivity to the obese maternal environment.

KEGG and GSEA enrichment analysis within each annotated E8.5 cell lineage revealed that common pathways and genes were dysregulated across multiple cell lineages (Supplementary Table 2, Supplementary Table 3). Glycosphingolipid biosynthesis and glycan degradation, cell junction organisation, and cell migration were among the most enriched pathways in upregulated DEGs. Cholesterol and lipid metabolism, lipoprotein particle metabolism, and vitamin transport were among the most enriched pathways in downregulated DEGs. These analyses reveal that maternal obesity alters embryonic transcriptional programs at the global level, such as structural and cell migratory pathways. Yet it also alters transcriptional programs in specific cell lineages, such as cholesterol and lipoprotein metabolism pathways, which were only detected once individual cell lineages were analysed.

### 3.3 Obesity during pregnancy alters chromatin accessibility specifically in embryonic and extraembryonic cell lineages

**Figure 3:**
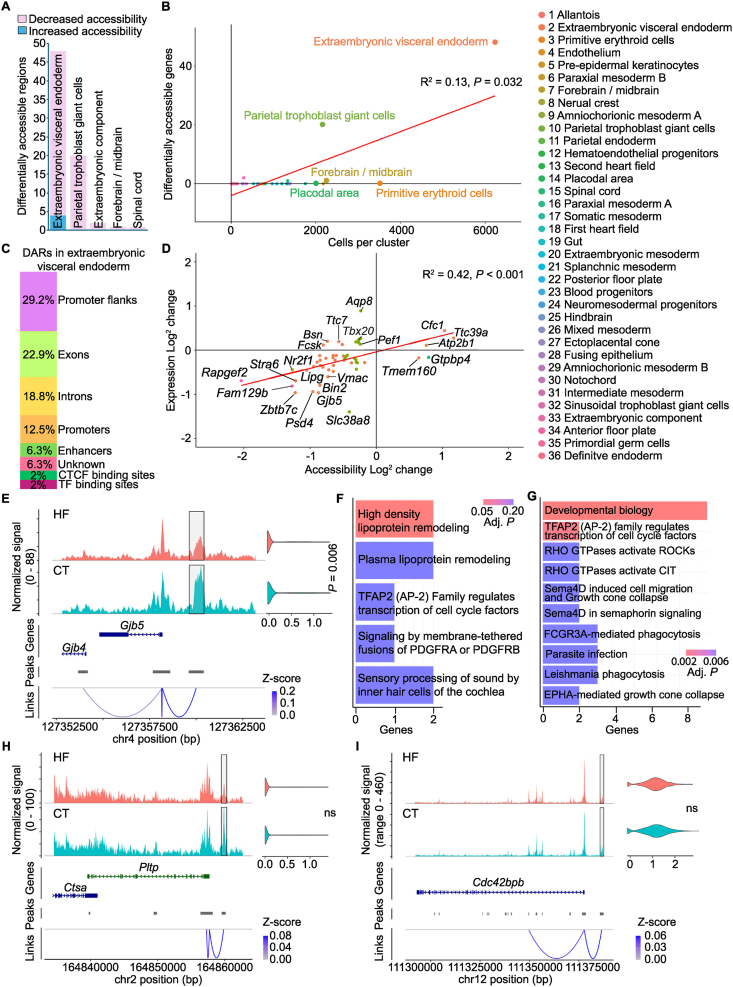
**Maternal high-fat diet alters chromatin accessibility in select embryonic and extraembryonic cell lineages. A**, Number of regions with different accessibility (y-axis) across cell lineages. **B**, Correlation between number of cells per cell lineage (x-axis) and number of differentially accessible genes per cell lineage (y-axis); Pearson's correlation coefficient R = 0.13, *P* = 0.032. Each dot represents a cell lineage, colored by cluster identity corresponding to distinct cell types (legend; right). **C**, Distribution of regions with increased or decreased accessibility across promoter flanks, exons, introns, promoters, enhancers, transcription factor binding sites, and unknown regions in HF extraembryonic visceral endoderm. **D**, Correlation between chromatin accessibility (x-axis) and expression (y-axis); Pearson's correlation coefficient R = 0.42, *P* < 0.001. Each dot represents a differentially accessible gene (DAG), colored by cluster identity corresponding to extraembryonic visceral endoderm, parietal trophoblast giant cells, extraembryonic component, forebrain/midbrain, or spinal cord (legend; right). **E**, snATAC-seq tracks and snRNA-seq violin plot of Gap junction protein beta 5 (*Gjb5*) in extraembryonic visceral endoderm in HF and CT E8.5 embryos. The grey box highlights the region that is significantly less accessible in HF E8.5 embryos. Links represent the correlation (Z-score) between peak accessibility and gene expression. **F**, Reactome pathway enrichment amongst DAGs in extraembryonic visceral endoderm of HF E8.5 embryos. Terms are coloured and organized based on adjusted *P-*values, with lower *P-*values at the top. Genes refers to the number of query genes that were detected in each Reactome pathway. **G**, Reactome pathway enrichment amongst DAGs in parietal trophoblast giant cells of HF E8.5 embryos. Terms are coloured and organized based on adjusted *P-*values, with lower *P-*values at the top. Genes refers to the number of query genes that were detected in each Reactome pathway. **H**, snATAC-seq tracks and snRNA-seq violin plot of Phospholipid transfer protein (*Pltp*) in extraembryonic visceral endoderm in HF and CT E8.5 embryos. Grey box highlights the region that is significantly less accessible in HF E8.5 embryos. Links represent the correlation (Z-score) between peak accessibility and gene expression. **I**, snATAC-seq tracks and snRNA-seq violin plot of CDC42 binding protein kinase beta (*Cdc42bpb*) in parietal trophoblast giant cells in HF and CT E8.5 embryos. Grey box highlights the region that is significantly less accessible in HF E8.5 embryos. Links represent the correlation (Z-score) between peak accessibility and gene expression.

To identify epigenetic alterations that could contribute to transcriptional dysregulation in response to obesity during pregnancy, we analyzed chromatin accessibility across all E8.5 lineages in HF and CT embryos. Chromatin accessibility was significantly different only in extraembryonic visceral endoderm, parietal trophoblast giant cells, extraembryonic component, spinal cord, and forebrain/midbrain lineages in HF embryos (Figure 3A, Supplementary Table 4). As in the transcriptional analyses, the number of differentially accessible regions (DARs) did not scale directly with lineage size (Figure 3B). Extraembryonic visceral endoderm and parietal trophoblast giant cells contained the most DARs, whereas several large lineages, including primitive erythroid cells, pre-epidermal keratinocytes, and placodal area, contained none. Thus, maternal obesity alters chromatin accessibility in a subset of lineages, suggesting chromatin-level responses that are sensitive to the obese maternal environment.

Changes in chromatin accessibility within cis-regulatory regions containing transcription factor binding sites can influence transcription [50]. To assess the transcriptional consequences of altered accessibility, we assigned each DAR to its nearest gene and then examined whether these differentially accessible genes (DAGs) were also differentially expressed. Most DAGs did not significantly change expression, although there was a positive correlation between chromatin accessibility change and expression (Figure 3D). Extraembryonic visceral endoderm had forty-eight DAGs, and although the DAR were in regulatory regions (Figure 3C), only two DAGs responded transcriptionally in HF embryos. Gap junction protein beta 5 (*Gjb5*) expression and accessibility at its promoter were significantly decreased (Figure 3E), and ATPase Ca^2+^ transporting plasma membrane 1 (*Atp2b1*) expression and accessibility at an upstream enhancer were significantly increased (Supplementary Fig. 4C). Expression of *Gjb5* is specific to extraembryonic tissues in the post-implantation mouse embryo [51], and its expression is critical for the differentiation of trophoblast stem cells within the ectoplacental cone into placental trophoblast subpopulations [52]. *Atp2b1* encodes a member of the plasma membrane calcium transporters, which are key in cell migration, differentiation, and signal transduction [53]. However, the remainder extraembryonic visceral endoderm DAGs and all DAGs in parietal trophoblast giant cells, extraembryonic component, spinal cord, and forebrain or midbrain remained transcriptionally unchanged.

Chromatin architecture often shifts before transcription changes [54,55]. Thus, many DAGs may mark loci where obesity induces regulatory changes that precede transcriptional dysregulation. Reactome pathway analysis of extraembryonic visceral endoderm DAGs identified high-density lipoprotein particle remodeling as the only enriched pathway (Figure 3F). The extraembryonic visceral endoderm mediates early nutrient transfer between mother and embryo [23,56]. HF reduced accessibility at regulatory elements of genes involved in lipoprotein transport, such as Phospholipid Transfer Protein (*Pltp*) [57] (Figure 3H). Suggesting that obesity during pregnancy alters the regulatory landscape that controls lipoprotein transport during early development. Reactome pathway analysis of parietal trophoblast giant cell DAGs enriched for pathways related to RHO GTPase activity, cell migration, and growth cone dynamics (Figure 3G). Parietal trophoblast giant cells invade the decidua and maternal capillaries during implantation and early placental growth [58]. HF induced decreased accessibility at promoters of genes that maintain cell shape and migration, such as CDC42 binding protein kinase beta (*Cdc42bpb*) [59] (Figure 3I). Thus, obesity during pregnancy affects the chromatin landscape of genes that support trophoblast motility, potentially influencing how these cells interact with decidual tissues during placental development.

### 3.4 Accessibility of DARs enriched for STAT, estrogen receptor, and retinoic acid receptor binding motifs is altered in specific extraembryonic lineages

Transcription factors and co-factors interactively bind regulatory regions within accessible chromatin to control gene expression [60]. To identify transcription factor activities that may have been affected in embryos exposed to a HF maternal diet, we performed motif enrichment analysis on DARs in extraembryonic visceral endoderm and parietal trophoblast giant cells.

In parietal trophoblast giant cells, DARs were enriched for motifs recognized by ESRRG and STAT4 (Table 2). These transcription factors regulate trophoblast energy metabolism and invasive behavior during differentiation, processes that support early placentation [61]. During implantation, low oxygen conditions and decidua-derived cytokines regulate ESRRG activity and promote transcriptional programs involved in mitochondrial biogenesis and energy metabolism [[62], [63], [64]]. The JAK–STAT pathway regulates genes involved in migration and invasion in trophoblast cells [[61], [62], [63]]. Embryos exposed to a HF maternal diet exhibited reduced accessibility at ESRRG- and STAT4-associated motifs. This pattern suggested that accessibility of regulatory elements supporting trophoblast motility and metabolic programs may have been affected in parietal trophoblast giant cells of HF embryos.

**Table 2:** Enriched motifs in extraembryonic visceral endoderm and parietal trophoblast giant cell differential accessible regions (DARs).

Cell lineage	JASPAR Motif ID	Motif name
Extraembryonic visceral endoderm	MA0065.2	Pparg::Rxra
	MA0677.1	Nr2f6
	MA0512.2	Rxra
	MA0521.1	Tcf12
	MA1619.1	Ptf1a(var.2)
	MA0857.1	Rarb
	MA0493.1	Klf1
	MA1627.1	Wt1
	MA0816.1	Ascl2
	MA0591.1	Bach1::Mafk
	MA1604.1	Ebf2
	MA1628.1	Zic1::Zic2
	MA1622.1	Smad2::Smad3
	MA1615.1	Plagl1
	MA0859.1	Rarg
	MA1616.1	Prdm15
	MA1630.1	Znf281
	MA0742.1	Klf12
	MA0125.1	Nobox
	MA0505.1	Nr5a2
	MA0002.2	RUNX1
	MA0150.2	Nfe2l2
	MA0898.1	Hmx3
	MA1623.1	Stat2
	MA0006.1	Ahr::Arnt
	MA0146.2	Zfx
	MA0603.1	Arntl
	MA0720.1	Shox2
	MA0676.1	Nr2e1
	MA1629.1	Zic2
	MA0832.1	Tcf21
	MA1621.1	Rbpjl
	MA0709.1	Msx3
	MA1620.1	Ptf1a(var.3)
	MA0897.1	Hmx2
	MA0604.1	Atf1
	MA1684.1	Foxn1
Parietal trophoblast giant cells	MA0518.1	Stat4
	MA0643.1	Esrrg

Consistent with reduced expression of retinol transporters across many lineages in HF embryos, DARs in extraembryonic visceral endoderm enriched for motifs recognized by retinoic acid receptors and retinoid X receptors (Table 2). Retinol enters cells through the RBP-dependent pathway and the retinyl-ester-containing lipoprotein pathway [65], retinoids are then converted into retinoic acid. Retinoic acid interacts with RAR and RXR receptors to regulate spatial and temporal transcriptional programs during embryogenesis [66]. Embryos exposed to a HF maternal diet displayed reduced accessibility at motifs recognized by RXRA, RARG, RARB and PPARG::RXRA within extraembryonic visceral endoderm. This reduction suggests that the function of regulatory elements involved in retinoic acid signaling may have been affected in this lineage.

**Figure 4:**
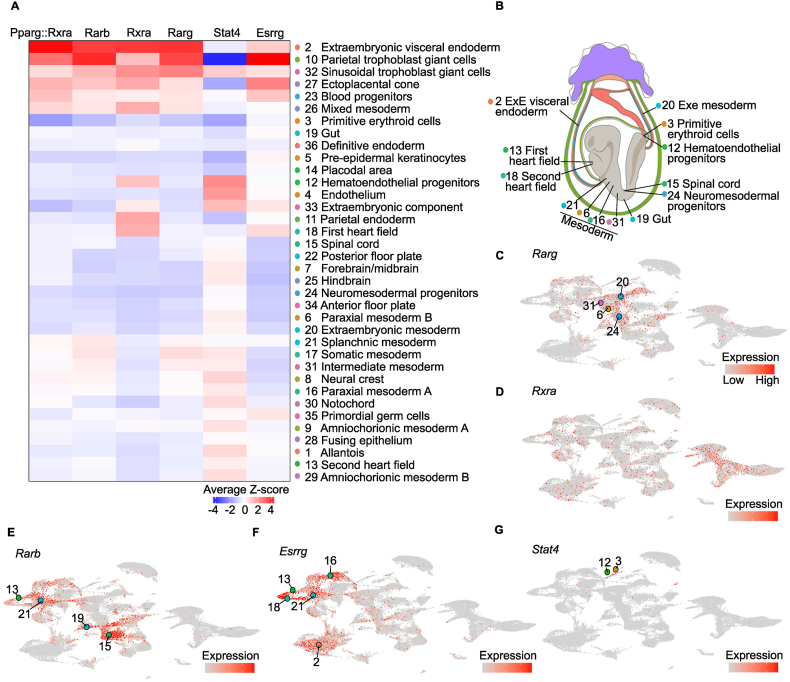
**DAR and accessible chromatin regions are enriched in Esrrg and retinoic acid receptor motifs in E8.5 extraembryonic visceral endoderm and parietal trophoblast giant cells. A,** Heatmap of average chromVAR Pparg:Rxra, Rarb, Rxra, Rarg, Stat4, and Esrrg enrichment (x-axis) across all annotated cell lineages (y-axis). Box color indicates average motif enrichment across all accessible chromatin regions within each cell lineage. Negative values indicate lower than average enrichment, and positive values indicate higher than average enrichment. Dot color next to each cell lineage indicates cluster identity as in Figure 1B–C. **B**, Schematic of E8.5 embryo highlights cell lineages in which *Rarg, Rxra, Rarb, Esrrg*, and *Stat4* are highly expressed according to **C-G**. **C-G**, Expression of *Rarg*, *Rxra*, *Rarb*, *Esrrg*, *and Stat4* across UMAP embeddings of all nuclei profiled by snRNA-seq. Dot color and label numbers indicate cluster identity, as in **A-B**.

A complex network of transcription factors governs lineage specification during organogenesis [19]. To assess how the factor binding motifs enriched in DARs relate to broader E8.5 regulatory programs, we calculated per-cell motif activity scores using chromVar [67]. Accessible chromatin across lineages enriched for multiple transcription factors at E8.5 (Supplementary Fig. 5A). Accessible chromatin in extraembryonic visceral endoderm and parietal trophoblast giant cells displayed strong enrichment for motifs recognized by RXRA, RARG, RARB, PPARG::RXRA and ESRRG (Figure 4A). Expression of these receptors was enriched within mesodermal, neural, and cardiac cell lineages (Figure 4B–F). In contrast, accessible chromatin in these lineages did not enrich for STAT4 motifs, and *Stat4* expression was not enriched in any cell lineage (Figure 4G). Since chromatin accessibility and transcriptional output can be asynchronous [55], the mismatch between motif enrichment and TF expression suggests that these TFs could function in extraembryonic visceral endoderm and parietal trophoblast giant cell development beyond E8.5. Thus, accessibility of regulatory regions linked to transcription factors that govern trophoblast motility, energy metabolic programs, and retinoic acid signaling is predominantly altered in extraembryonic lineages exposed to a HF maternal diet.

### 3.5 Lipoprotein and retinoid metabolism are specifically affected in extraembryonic visceral endoderm of embryos exposed to a HF maternal diet

**Figure 5:**
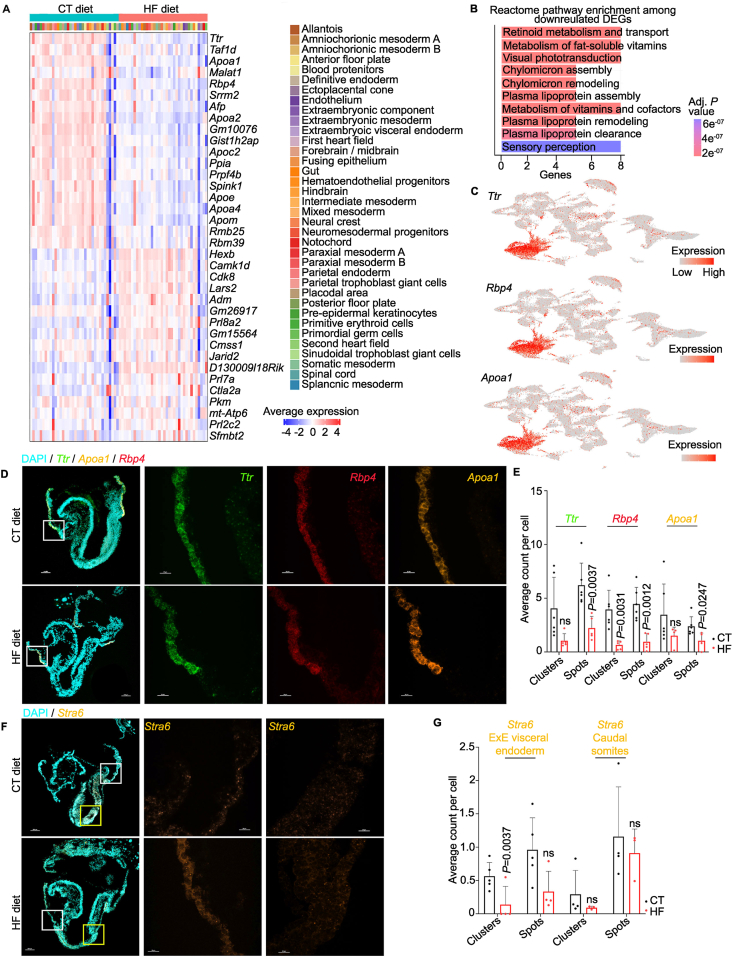
**Maternal high-fat diet alters the expression of lipoprotein and retinoid transporters in E8.5 extraembryonic visceral endoderm. A**, Heatmap of average expression of the most upregulated and downregulated genes (y-axis) across all annotated cell lineages. Genes are clustered according to diet. The color scale indicates average expression, negative values indicate lower than average expression, and positive values indicate higher than average expression. **B**, Reactome pathway enrichment amongst genes that were consistently downregulated in HF E8.5 embryos. Terms are coloured and organized based on adjusted *P-*values, with lower *P-*values at the top. Genes refers to the number of query genes that were detected in each Reactome pathway. **C**, Expression of *Ttr*, *Rbp4*, and *Apoa1* across UMAP embeddings of all nuclei profiled by snRNA-seq. **D**, RNA immunofluorescence staining for *Ttr* (green), *Rbp4* (red), and *Apoa1* (orange) in E8.5 whole embryo sections from CT and HF dams. The white boxes correspond to close-ups. First panel magnification: 10x; scale bar 100 μm. *Ttr* (green), *Rbp4* (red), and *Apoa1* (orange) panel magnifications: 60x; scale bar 20 μm. **E**, Quantification of individual mRNA transcripts (spots) and clusters of mRNA transcripts (clusters) per cell confirmed significantly reduced expression of *Ttr*, *Rbp4*, and *Apoa1* in E8.5 extraembryonic visceral endoderm of embryos of HF mice. HF N = 5, CT N = 5. **F**, RNA immunofluorescence staining of *Stra6* (orange) in E8.5 whole embryos from CT and HF dams. The white boxes correspond to close-ups. First panel magnification: 10x; scale bar 100 μm. *Stra6* panel magnifications: 60x; scale bar 20 μm. **G**, Quantification of individual mRNA transcripts (spots) and clusters of mRNA transcripts (clusters) per cell confirmed significantly reduced expression of Stra6 in E8.5 extraembryonic visceral endoderm of embryos of HF mice. HF N = 4, CT N = 5.

Gene expression analyses across individual E8.5 lineages revealed unique expression changes in a subset of lineages in embryos exposed to a HF maternal diet. In addition to these lineage-specific effects, we identified a set of genes that were consistently dysregulated across many lineages. Seventeen genes were upregulated and nineteen genes were downregulated in at least a quarter of all detected lineages (Table 3). Expression of these genes increased or decreased in every HF lineage examined (Figure 5A). This pattern indicated that embryos exposed to a HF maternal diet mounted both lineage-specific and shared transcriptional responses.

**Table 3:** Top consistently upregulated genes across multiple cell lineages.

Gene	Number of affected cell lineages	Status in HF embryo
*Ttr*	18	Down
*Taf1d*	16	Down
*Apoa1*	15	Down
*Malat1*	14	Down
*Srrm2*	14	Down
*Rbp4*	14	Down
*Afp*	12	Down
*Hist1h2ap*	12	Down
*Gm10076*	12	Down
*Apoa2*	12	Down
*Apoc2*	11	Down
*Prpf4b*	10	Down
*Ppia*	10	Down
*Spink1*	10	Down
*Apoe*	9	Down
*Rbm39*	8	Down
*Rbm25*	8	Down
*Apom*	8	Down
*Apoa4*	8	Down
*Hexb*	27	Up
*Camk1d*	26	Up
*Cdk8*	25	Up
*Lars2*	23	Up
*Adm*	22	Up
*Gm26917*	21	Up
*Prl8a2*	19	Up
*Gm15564*	18	Up
*Cmss1*	16	Up
*Jarid2*	16	Up
*Prl7a1*	15	Up
*D130009I18Rik*	15	Up
*mt-Atp6*	10	Up
*Pkm*	10	Up
*Ctla2a*	10	Up
*Sfmbt2*	9	Up
*Prl2c2*	9	Up

Consistently, downregulated genes enriched for Reactome pathways related to retinoid metabolism, retinoid transport, chylomicron assembly and plasma lipoprotein assembly (Figure 5B). Genes driving these enrichments included several apolipoproteins, as well as *Rbp4* and *Ttr*, which mediate transport of lipoproteins, retinol, and thyroxine from the mother to the embryo [65,68]. Downregulation of these genes, across many lineages suggested that nutrient transport processes could be broadly affected in embryos exposed to a HF maternal diet.

Reduced expression of *Rbp4*, *Ttr* and multiple apolipoproteins throughout the embryo, together with reduced chromatin accessibility at retinoic acid receptor response elements in extraembryonic visceral endoderm (Table 2), suggests that retinoic acid signaling may have been altered in embryos exposed to a HF maternal diet due to decreased retinol delivery. To investigate this, we performed fluorescent RNA *in situ* hybridization for genes that respond to retinoic acid signaling [69]. Expression of *Ttr*, *Rbp4,* and *Apoa1* was reduced in extraembryonic visceral endoderm of HF embryos (Figure 5D,E). At E8.5, these transporters are normally expressed at high levels in the visceral yolk sac (Figure 5C), which mediates nutrient transfer before placental development [23,65]. These observations suggest that retinol delivery through the visceral yolk sac to the embryo was reduced in HF embryos.

We next asked whether reduced expression of retinoid transport genes in HF embryos accompanied changes in retinoic acid receptor expression. Expression of *Rara*, *Rarb*, and *Rarg* did not differ between CT and HF embryos in either the caudal somite region or the forebrain (Supplementary Fig. 5B–E), where these receptors are highly expressed [70]. This indicates that the effects of maternal HF exposure were selective within the retinoid pathway, affecting genes involved in retinol transport but not the receptor genes themselves. In contrast, *Stra6*, which encodes the principal receptor responsible for retinol uptake [71], was significantly reduced only in the extraembryonic visceral endoderm of HF embryos (Figure 5F–G). Together, these findings suggest that maternal HF exposure primarily disrupts retinoid transport and uptake in extraembryonic visceral endoderm.

Thus, transcriptional programs governing lipoprotein transport and retinoid metabolism are predominantly altered in extraembryonic visceral endoderm of embryos exposed to a HF maternal diet, identifying this lineage as a key site where early nutrient-handling pathways are affected.

## 4 Discussion

Obesity during pregnancy is associated with congenital anomalies [14] and long-term cardiometabolic risk in offspring [5], yet the earliest responses to an obesogenic intrauterine environment remain unknown. Here, transcriptional and chromatin accessibility mapping in single nuclei revealed preserved cell lineage allocation in embryos of obese dams. However, transcription was widely dysregulated, with broad suppression of oxidative phosphorylation and upregulation of pathways involved in signal transduction, cytoskeleton remodeling, and cell migration (Figure 1E–F, Supplementary Fig. 4A and B). This suggests that maternal obesity perturbs core metabolic and morphogenetic programs during early development when progenitors of multiple cell lineages are rapidly expanding and differentiating. While gene dysregulation was widespread, chromatin accessibility changed primarily in extraembryonic visceral endoderm and parietal trophoblast giant cells, with limited changes in the broader extraembryonic component, spinal cord, and forebrain/midbrain lineages (Figure 3, Supplementary Fig. 4C). Chromatin accessibility changes can precede measurable transcriptional shifts, consistent with chromatin priming that forecasts later gene regulation [55]. Thus, chromatin accessibility changes in embryos of obese dams could represent early regulatory events that later contribute to embryonic gene dysregulation.

At E8.5 the yolk sac mediates nutrient transport before placental maturation [72]. Our findings of predominant gene expression and chromatin accessibility changes in extraembryonic lineages suggest that the maternal–fetal interface is particularly vulnerable, with extraembryonic visceral endoderm and trophoblast lineages as potential first responders to maternal metabolic disturbance. Consistent with this, genes involved in retinol and lipoprotein transport were downregulated across multiple lineages (Figure 5A). In addition, expression of *Apoa1*, *Rbp4*, and *Ttr* was reduced in the extraembryonic visceral endoderm of HF embryos, and *Stra6* was significantly reduced only in this lineage (Figure 5D–G). Because the visceral endoderm mediates retinol uptake [25,26,73], these findings suggest that maternal HF exposure may impair vitamin A delivery and processing through extraembryonic tissues.

Differentially accessible regions in extraembryonic visceral endoderm were enriched for retinoic acid receptor and RXR-related motifs (Table 2). Because these motifs are predicted to contribute to regulatory programs in extraembryonic visceral endoderm and parietal trophoblast giant cells (Figure 4A), these findings suggest that maternal HF exposure could perturb retinoid-associated gene regulation in extraembryonic tissues. Retinoic acid signaling coordinates patterning and organogenesis; and perturbing it causes broad congenital phenotypes [70,74]. Chromatin accessibility changes in parietal trophoblast giant cells were enriched for motifs linked to ESRRG and STAT family signaling. ESRRG regulates trophoblast energy metabolism and mitochondrial programs [61,75], and JAK STAT signaling has been linked to trophoblast function and invasion [63,76]. These findings argue that beyond a generalized embryonic stress response, obesity during pregnancy impacts on specific extraembryonic regulatory circuits governing nutrient transport and trophoblast behavior. Moreover, our results suggest that metabolic shifts in an obesogenic *in utero* environment converge on disrupted retinoid and lipoprotein handling at the maternal–fetal interface, accompanied by broad embryonic metabolic remodeling.

Global suppression of oxidative phosphorylation genes in embryos exposed to maternal obesity aligns with evidence that maternal obesity can rewire metabolic and developmental gene networks in cardiac progenitor cells [15]. Our atlas extends this concept beyond a single organ lineage by showing that transcriptional programs consistent with reduced respiratory capacity and altered cytoskeletal and migratory states emerge in diverse lineages during early organogenesis (Supplementary Table 2). One interpretation is that altered maternal substrates and oxygen tension shift energetic constraints during a period of rapid morphogenesis, leading to compensatory or maladaptive transcriptional remodeling. An alternative, not mutually exclusive, possibility is that impaired extraembryonic nutrient handling reduces delivery of lipophilic metabolites and morphogen cofactors, indirectly reshaping embryonic transcriptional states.

While single-nucleus analysis covers multiple cell lineages, it captures a fraction of total cellular RNA and accessible chromatin compared to bulk approaches. Thus, the transcriptional and regulatory differences identified here likely underrepresent the molecular effects induced in the embryo by obesity during pregnancy. Thus, the restricted set of chromatin accessibility changes observed in embryonic and extraembryonic lineages may underestimate the extent to which regulatory architecture is perturbed across the embryo. Moreover, metabolic and signaling perturbations associated with maternal obesity may influence post-transcriptional regulation, protein stability, metabolite availability, or signaling pathway activity, none of which are directly captured by single-nucleus Multiome. Despite technical constraints, our analysis detected coherent dysregulation of nutrient transport pathways, retinoid handling, and oxidative metabolism, particularly at the maternal–fetal interface. This suggests that these pathways represent some of the most robust embryonic responses to maternal obesity. Future studies, including additional developmental stages and directly measuring maternal and embryonic retinoid or lipoprotein abundance, are required to assess the effects of altered nutrient transport in embryonic cell state transitions.

These findings suggest that an obesogenic *in utero* environment impacts primarily on extraembryonic tissues, particularly the extraembryonic visceral endoderm, where it alters regulatory DNA accessibility and suppresses retinoid and lipoprotein transport programs. This extraembryonic vulnerability coincides with broad embryonic transcriptional remodeling, including repression of oxidative phosphorylation and activation of hypoxia and morphogenesis-related pathways. Together, these findings point to nutrient transport and its transcriptional regulation at the maternal–fetal interface as a potential mechanism for the influence of maternal obesity on organogenesis and long-term developmental outcomes.

## CRediT authorship contribution statement

**Amalia Caballero:** Writing – review & editing, Writing – original draft, Validation, Methodology, Investigation, Formal analysis, Data curation. **Lijun Chi:** Methodology, Investigation, Formal analysis. **Paul Delgado-Olguín:** Writing – review & editing, Writing – original draft, Validation, Supervision, Project administration, Funding acquisition, Formal analysis, Data curation, Conceptualization.

## Funding

This work was supported by the 10.13039/501100000024Canadian Institutes of Health Research, grant numbers 162208, 149046, and 468633; and the 10.13039/100004411Heart & Stroke Foundation of Canada, grant number G-17-0018613.

## Declaration of competing interest

The authors declare the following financial interests/personal relationships which may be considered as potential competing interests: Paul Delgado Olguin reports financial support was provided by Canadian Institutes of Health Research. Paul Delgado Olguin reports financial support was provided by Heart and Stroke Foundation of Canada. If there are other authors, they declare that they have no known competing financial interests or personal relationships that could have appeared to influence the work reported in this paper.

## Data Availability

Sequencing data been deposited in Gene Expression Omnibus (GEO) under the accession number GSE327799.
