# Supplementary material for: Maternal obesity remodels nutrient transport transcriptional programs in early mouse embryonic and extraembryonic cell lineages
Source: Mol Metab. 2026 Apr 30;108:102375. doi: 10.1016/j.molmet.2026.102375 (PMC13188144; doi:10.1016/j.molmet.2026.102375)
Supplement: Multimedia component 1 [file mmc1.pdf]

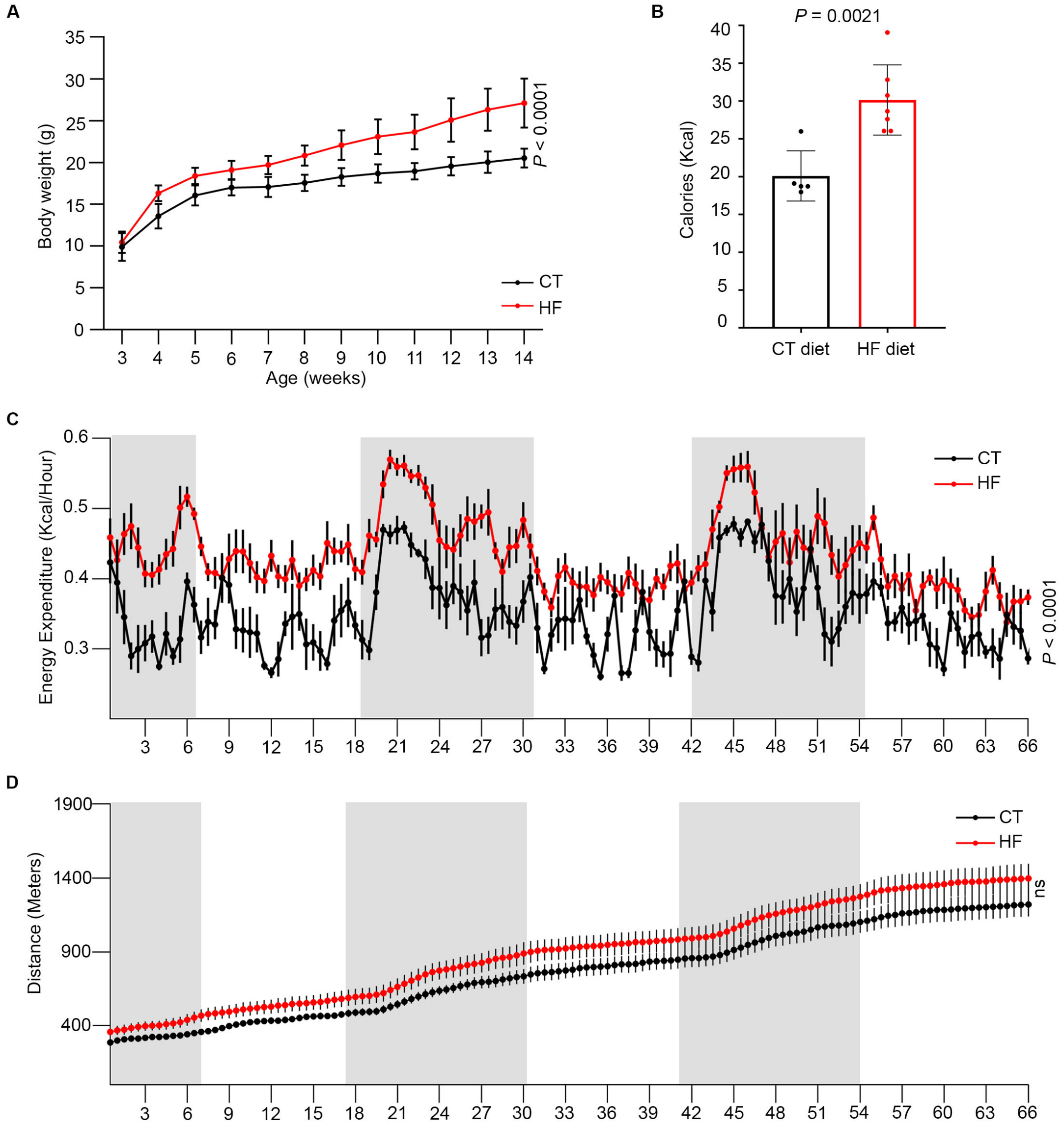

Supplementary Figure 1. A high-fat diet alters female mice metabolism.
A, Weight gain of female mice fed a control (CT) or high-fat (HF) diet. Data was analysed using a mixed-effect model of the form y ~ diet + week + (1 | mice) with multiple comparison and Sidak correction. Error bars denote the mean ± SD; CT n = 26 and HF n = 28. **B**, Caloric consumption of 14-week-old CT and HF female mice during the 4 days they spent in Promethion metabolic cages. Data was analysed using an unpaired t-test. Error bars denote the mean ± SD; CT n = 5 and HF n = 7. **C**, Energy expenditure measurements of 14-week-old CT and HF female mice during 4 days in the metabolic cages. **D**, Measurements of traveled distances of 14-week-old CT and HF female mice during 4 days in the metabolic cages. Promethion metabolic cage data was analysed using two-way ANOVA with multiple comparison and Sidak correction. Error bars denote the mean ± SEM; CT n = 5 and HF n = 7. Dark boxes represent nighttime for both **C** and **D**.

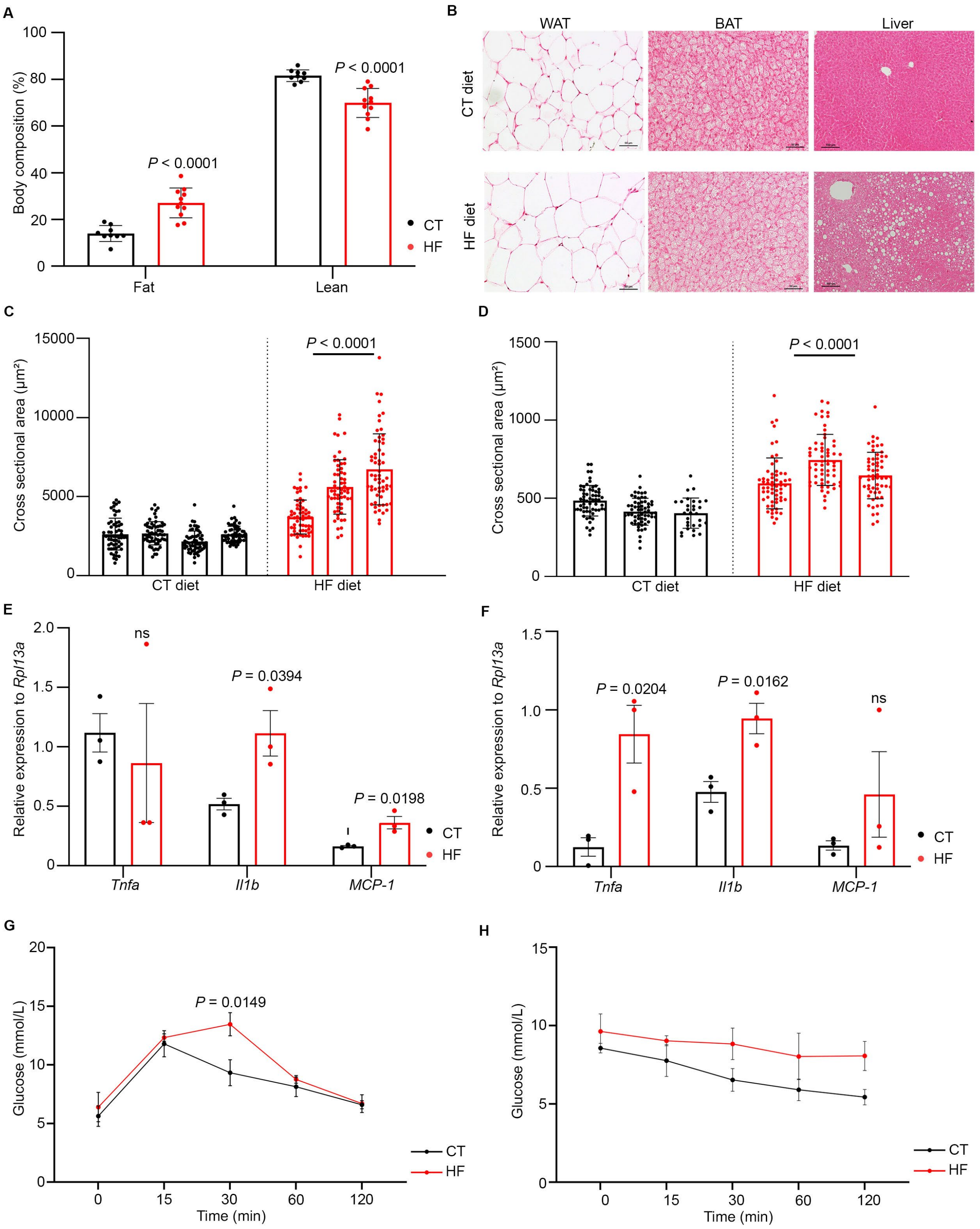

Supplementary Figure 2. A high-fat diet induces obesity in female mice without overt diabetes or hyperglycemia.

A, Body composition of CT- and HF-fed female mice after 10 weeks on the diet. Data was analysed using unpaired t-tests. Error bars denote the mean ± SD; CT n = 9 and HF n = 11. **B**,

Representative H & E staining of perigonadal white adipose tissue (WAT), supraspinal brown adipose tissue (BAT), and liver of CT- and HF-fed female mice after 12 weeks on the diet. HF mice had diffuse micro (small white dots) and macro (large white dots) vesicular steatosis. Magnification of adipose sections: 20x; scale bar 50μm. Magnification of liver sections: 10x, scale bar 100μm. **C**, Quantification of cell surface area of 60 randomly chosen white adipocytes per sample. Error bars denote the mean ± SD; CT n = 4 and HF n = 3. **D**, Quantification of cell surface area of 60 randomly chosen brown adipocytes per sample. Error bars denote the mean ± SD; CT n = 3 and HF n = 3. Two-way ANOVA tests were conducted to determine differences in adipocyte surface area. **E**, RT-qPCR of tumor necrosis factor α (*Tnfα*), Interleukin 1 beta (*Il1b*), and Monocyte marker monocyte chemoattractant protein 1 (*MCP-1*) in supraspinal BAT of 16-week-old CT and HF female mice. **F**, RT-qPCR of *Tnfα*, *Il1b*, *MCP-1* in livers of 16-week-old CT and HF female mice. *Rpl13a* was used as reference. RT-qPCR data was analysed using unpaired t-tests. Error bars denote the mean ± SEM; CT n = 3 and HF n = 3. **G**, Glucose tolerance test of CT- and HF-fed female mice after 13 weeks of diet. **H**, Insulin tolerance test of CT- and HF-fed female mice after 13 weeks of diet. Glucose and insulin tolerance data was analysed using two-way ANOVA tests. Error bars denote the mean ± SD; CT n = 3 and HF n = 3.

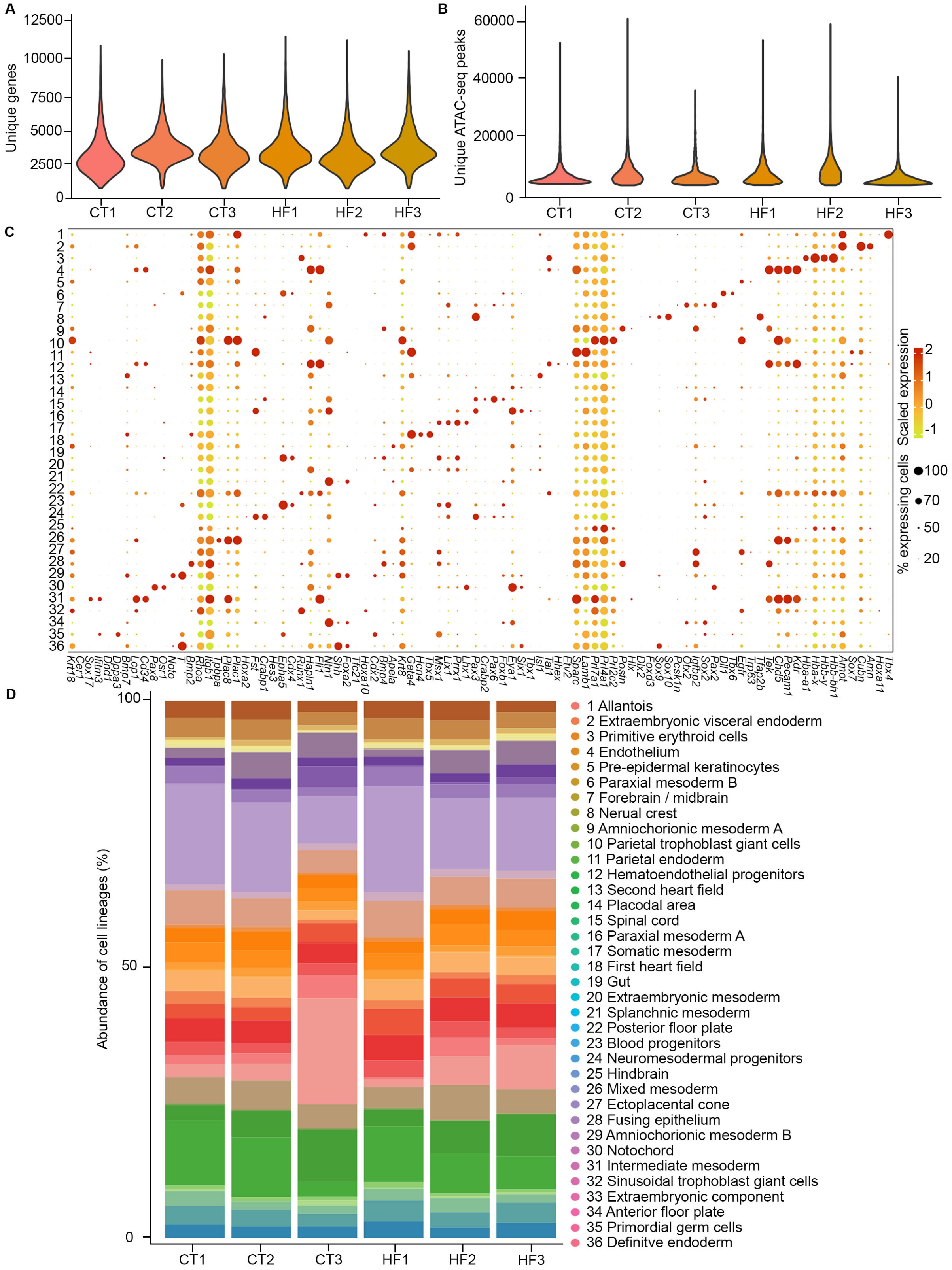

Supplementary Figure 3. Maternal obesity does not overtly disrupt lineage allocation or progenitor differentiation in E8.5 cell states.

A, Violin plots depicting gene detection between biological samples (x-axis). **B**, Violin plots depicting ATAC-seq peak detection between biological samples (x-axis). **C**, Dot plot showing scaled expression of canonical marker genes (x-axis) across all cell clusters (y-axis). Dot size represents the percentage of nuclei expressing each gene, and dot color indicates the average-scaled expression level. Marker gene expression validates cluster identity assignments and highlights transcriptional signatures of major embryonic lineages. **D**, Bar plot illustrating the proportion of annotated cell lineages captured in each sample (x-axis).

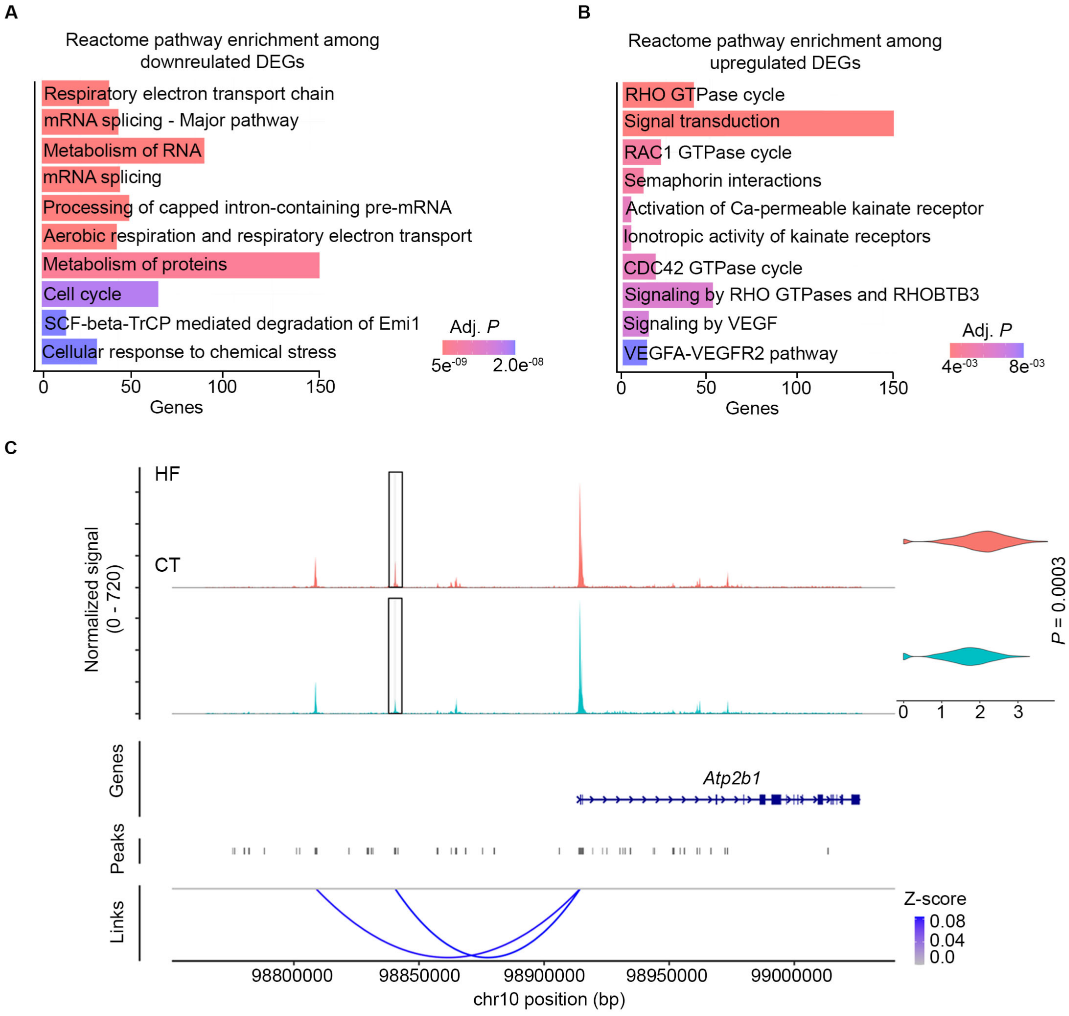

Supplementary Figure 4. Maternal high-fat diet alters metabolic, signaling, and migration programs at the transcription and chromatin accessibility level.

A, Reactome pathway enrichment amongst genes downregulated in HF E8.5 embryos. Terms are coloured and organized based on adjusted *P*-values, with lower *P*-values at the top. Genes refers to the number of query genes that were detected in each GO term. **B**, Reactome pathway enrichment amongst genes upregulated in HF E8.5 embryos. Terms are coloured and organized based on adjusted *P*-values, with lower *P*-values at the top. Genes refers to the number of query genes that were detected in each GO term. **C**, snATAC-seq tracks and snRNA-seq violin plot of ATPase Ca2+ transporting plasma membrane 1 (*Atp2b1*) in parietal trophoblast giant cells in HF and CT E8.5 embryos. The boxes highlight the region that was significantly more accessible in HF E8.5 embryos. Links represent the correlation (Z-score) between peak accessibility and gene expression.

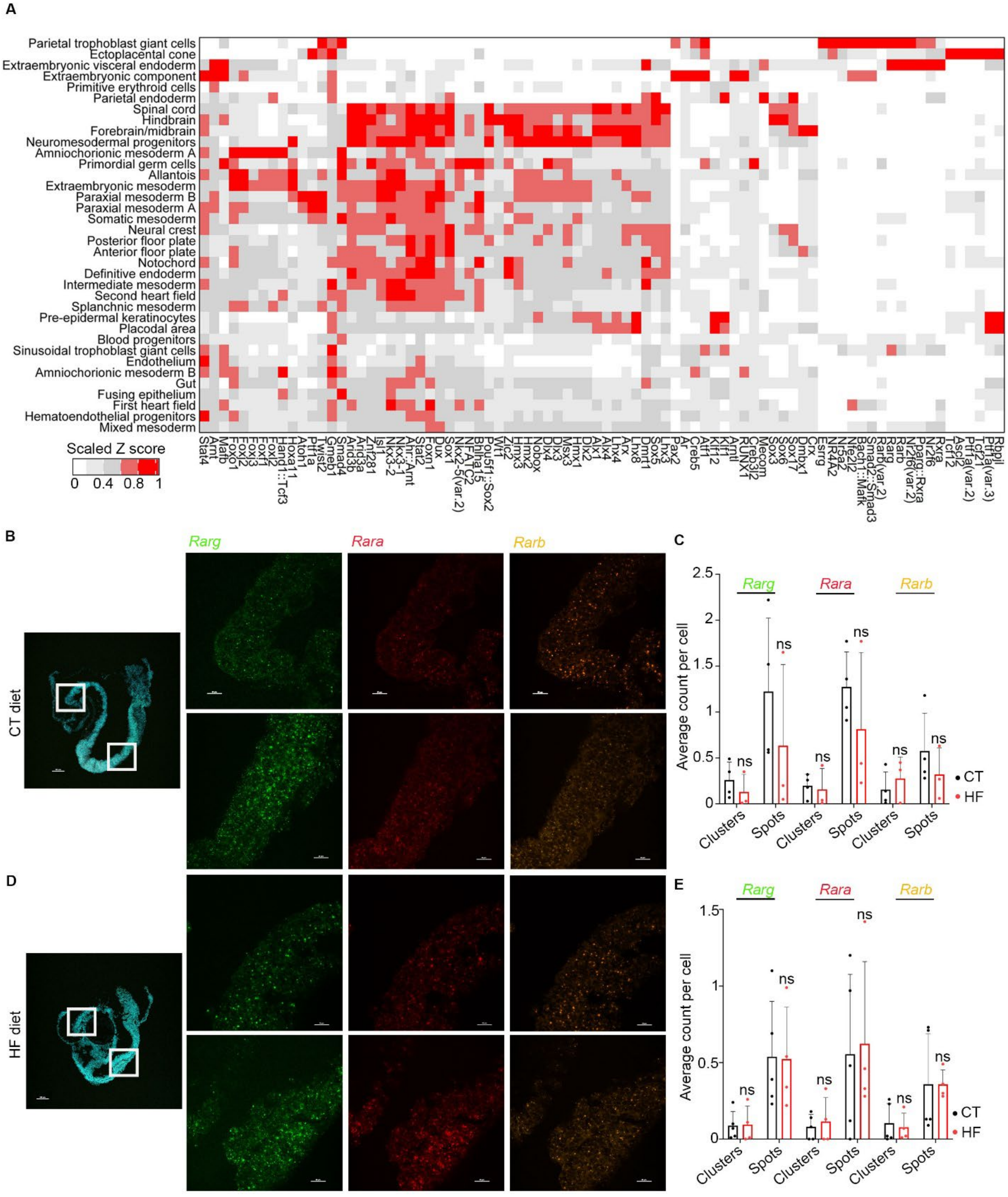

Supplementary Figure 5. Maternal high-fat diet does not alter expression of retinoic acid receptors in E8.5 embryos.

A, Heatmap of average chromVAR transcription factor binding motif enrichment (x-axis) across all annotated cell lineages (y-axis). Box color indicates average motif enrichment across all accessible chromatin regions within each cell lineage. Negative values indicate lower than average enrichment, and positive values indicate higher than average enrichment. Average

chromVAR score is scaled to highlight cell lineages with the highest enrichment of transcription factor binding motifs. **B**, RNA fluorescent *in situ* hybridization of *Rarg* (green), *Rara* (red), and *Rarb* (yellow) in sections of E8.5 embryos from CT dams. White boxes denote close-ups of the forebrain and caudal somite region. Scale bar in embryo image = 100 µm, close-ups = 20 µm. **C**, Quantification of individual mRNA transcripts (spots) and clusters of mRNA transcripts (clusters) per cell. CT n = 4 and HF n = 3. **D**. RNA fluorescent *in situ* hybridization of *Rarg* (green), *Rara* (red), and *Rarb* (yellow) in sections of E8.5 embryos from HF dams. White boxes denote close-ups of the forebrain and caudal somite region. Scale bar in embryo image = 100 µm, close-ups = 20 µm. **E**, Quantification of individual mRNA transcripts (spots) and clusters of mRNA transcripts (clusters) per cell, CT n = 4 and HF n = 3.
