# Supplementary material for: Maternal obesity remodels nutrient transport transcriptional programs in early mouse embryonic and extraembryonic cell lineages
Source: Mol Metab. 2026 Apr 30;108:102375. doi: 10.1016/j.molmet.2026.102375 (PMC13188144; doi:10.1016/j.molmet.2026.102375)
Supplement: Multimedia component 2 [file mmc2.docx]

**Supplementary Table 1. lineage-specific markers**

| **Cluster** | **Genes** |
| --- | --- |
| Allantois | *Tbx4, Hoxa11* |
| Extraembryonic visceral endoderm | *Amn, Cubn, Sox7, Amot* |
| Primitive erythroid cells | *Hbb-bh1, Hbb-y, Hba-x, Hba-a1* |
| Endothelium | *Kdr, Pecam1, Cdh5, Tek* |
| Pre-epidermal keratinocytes | *Tfap2b, Trp63, Egfr* |
| Paraxial mesoderm B | *Tbx6, Dll1* |
| Forebrain/Midbrain | *Pax2, Sox2, Igfbp2, Otx2, Pcsk1n* |
| Neural crest | *Sox10, Sox9, Foxd3, Dlx2* |
| Amniochorionic mesoderm A | *Hlx, Postn* |
| parietal trophoblast giant cells | *Prl2c2, Prl4a1, Prl7a1* |
| Parietal Endoderm | *Lamb1, Sparc* |
| Haematoendothelial progenitors | *Etv2, Hhex, Tal1* |
| Second heart field | *Isl1, Tbx1* |
| Placodal area | *Six1, Eya1* |
| Spinal cord | *Foxb1, Pax6, Crabp2* |
| Paraxial mesoderm A | *Pax3, Tbx1* |
| Somatic mesoderm | *Lhx1, Prrx1, Lix1, Msx1* |
| First heart field | *Tbx5, Hcn4, Gata4* |
| Gut | *Krt8, Apela* |
| Extraembryonic mesoderm | *Bmp4, Cdx2, Hoxa10* |
| Splanchnic mesoderm | *Tcf21, Isl1, Gata4* |
| Posterior floor plate | *Foxa2, Shh, Ntn1* |
| Blood progenitors | *Fli1, Hapln1, Runx1* |
| Neuromesodermal progenitors | *Cdx4, Epha5, Hes3* |
| Hindbrain | *Sox2, Crabp1, Fst, Pax2, Hoxa2* |
| Ectoplacental Cone | *Plac1, Plac8, Tpbpa* |
| Fusing epithelium | *Itgb1, Rhoa* |
| Amniochorionic mesoderm B | *Bmp2, Postn* |
| Notochord | *T, Noto* |
| Intermediate mesoderm | *Osr1, Lhx1, Pax2, Pax8* |
| Sinusoidal trophoblast giant cells | *Cd34, Cdh5, Lcp1, Pecam1* |
| Anterior floor plate | *Foxa2, Shh, Ntn1, Bmp7* |
| Primordial germ cells | *Dppa3, Dnd1, Ifitm3* |
| Definitive Endoderm | *Sox17, Cer1, Krt18* |

**Supplementary Table 2. Obesity during pregnancy leads to dysregulation of common pathways in multiple developing cell lineages**

| **System** | **Cell lineage** | **Structure** | **KEGG Pathways** | **GSEA** |
| --- | --- | --- | --- | --- |
| **Placenta and extraembryonic tissues** | Allantois | Umbilical cord | ˄ Glycosphingolipid biosynthesis and glycan degradation.  ˅ Cholesterol metabolism and lipid metabolism. | NA |
|  | Amniochorionic mesoderm A | Labyrinthine placenta | ˄ Glycosphingolipid biosynthesis and glycan degradation.  ˅ Cholesterol metabolism and PPAR signaling pathway. | NA |
|  | Amniochorionic mesoderm B | Labyrinthine placenta | ˄ Glycosphingolipid biosynthesis and glycan degradation. | ˅ Sensory perception and synaptic signaling. |
|  | Ectoplacental cone | Trophoblast stem cells | ˄ Aminoacyl-tRNA-biosynthesis and HIF1-signaling pathway.  ˅ Cholesterol metabolism and vitamin digestion and absorption | ˄ Cell junction assembly, cell junction organization, and cell adhesion.  ˅ High-density lipoprotein (HDL) particle remodeling. |
|  | Parietal trophoblast giant cells | Trophoblast giant cells that mediate endometrium and uterine arteries invasion | ˄ Mitophagy, oxidative phosphorylation, and gap junction.  ˅ Ribosome biogenesis. | ˄ Granzyme-mediated programmed cell death signaling and positive regulation of inflammatory response.  ˅ Ribosome biogenesis. |
|  | Sinusoidal trophoblast cells | Outermost layer of the interhaemal membrane of the labyrinth placenta | NA | ˄ Cell migration, cell-cell junction maintenance, and epithelial cell maturation.  ˅ Negative regulation of cytokine and mediator production. |
|  | Extraembryonic component | Cluster that expresses a combination of giant trophoblast and endothelial cell marker genes | NA | ˄ Axonogenesis and axon development.  ˅ Phospholipase C-activating G protein-coupled receptor signaling. |
|  | Extraembryonic mesoderm | Amnion, allantois, chorionic villi and visceral yolk sac | ˄ Glycosphingolipid biosynthesis and glycan degradation. | NA |
|  | Extraembryonic visceral endoderm | Visceral yolk sac | ˄ Mineral absorption and thyroid hormone signaling.  ˅ Oxidative phosphorylation. | ˄ Stimulus detection. |
|  | Parietal endoderm | Parietal yolk sac | ˄ Electron transport chain, oxidative phosphorylation, and ATP metabolic production. | NA |
| **Cardiovascular system** | First heart field | Myocardium of the left ventricle and portions of both atria | NA | ˄ Cell junction assembly and cell junction organization. |
|  | Second heart field | Myocardium of the right ventricle, portions of both atria, and outflow track | NA | NA |
|  | Endothelium | Innermost layer of all blood and lymphatic vessels | ˄ Glycosphingolipid biosynthesis and glycan degradation.  ˅ Cholesterol metabolism. | NA |
| **Hematopoietic system** | Hematoendothelial progenitors | Bipotent progenitor to both endothelial cells and primitive blood | ˅ Cholesterol metabolism and lipid metabolism. | ˄ Monoatomic ion transport. |
|  | Primitive erythroid cells | Nucleated primitive blood cells | ˄ Adherens junction.  ˅ Cholesterol metabolism. | ˅ Regulation of lipase activity, low-density lipoprotein particle remodeling, and High-density. lipoprotein (HDL) particle clearance. |
|  | Blood progenitors | Erythro-myeloid progenitors to both erythroid and myeloid cell lineages | NA | NA |
| **Nervous system** | Anterior floor plate | Governs the differentiation of anterior neural tube into forebrain, midbrain, and hindbrain. | NA | NA |
|  | Posterior floor plate | Governs the differentiation of posterior neural tube into spinal cord | NA | NA |
|  | Notochord | Rod-like structure along the anterior-posterior axis of the embryo, it serves as a signaling hub for neural tube, paraxial mesoderm, and gut differentiation | NA | NA |
|  | Forebrain/Midbrain | Precursors to the prosencephalon and the mesencephalon | ˅ Cholesterol metabolism, vitamin digestion and absorption, and fat digestion. | ˄ Immune and inflammatory response.  ˅ Lipoprotein particle remodeling, intestinal cholesterol absorption, and regulation of lipoprotein lipase activity. |
|  | Hindbrain | Precursor to the rhombencephalon | ˄ Glycosphingolipid biosynthesis and glycan degradation. | NA |
|  | Spinal cord | Cylindrical structure consisting of highly organized nerve bundles that carry motor and sensory signals | ˅ Cholesterol metabolism and PPAR signaling pathway. | ˄ Cell migration, cell motility, and locomotion.  ˅ Mitochondrial respiratory chain complex assembly. |
|  | Neural crest | Peripheral nervous system (PNS) | ˄ Glycosphingolipid biosynthesis and glycan degradation.  ˅ Necroptosis | NA |
|  | Placodal area | Cranial sensory organs such as the lens of the eye, the inner ear and the olfactory epithelium | ˄ Pentose phosphatase pathway and glycolysis/gluconeogenesis  ˅ Oxidative phosphorylation | ˄ Macrophage colony-stimulating factor signaling, myeloid leukocyte activation, and leukocyte proliferation. |
|  | Neuromesodermal progenitors | Derivatives of paraxial mesoderm and spinal cord | ˄ Glycosphingolipid biosynthesis and glycan degradation. | NA |
| **Respiratory and digestive system** | Definitive endoderm | Precursor to the respiratory and the digestive system. | ˅ Cholesterol metabolism, fat digestion and absorption, and PPAR signaling | NA |
|  | Splanchnic mesoderm | Splanchnic contributes to the development of vascular and muscular components of the gut, and it also contributes to the development of the mesenteries | NA | NA |
| **Urogenital system** | Intermediate mesoderm | Precursors to the definitive kidney (metanephros), the gonads (mesonephros), and connective ducts within both systems | NA | NA |
|  | Primordial germ cells | Gametes | NA | NA |
| **Musculoskeletal system** | Paraxial mesoderm A | Muscles, bones, and skin of the torso and the back (anterior) | ˅ Cholesterol metabolism. | ˄ Muscle filament sliding, cardiac muscle tissue morphogenesis, and neuromuscular process.  ˅ Triglyceride-rich lipoprotein particle remodeling and very-low-density lipoprotein particle remodeling. |
|  | Paraxial mesoderm B | Muscles, bones, and skin of the torso and the back (posterior) | ˅ Vitamin and fat digestion and absorption, cholesterol metabolism, and PPAR signaling. | ˄ Endothelial cell proliferation and cellular response to estradiol stimulus |
|  | Somatic mesoderm | ventral muscles, pelvic and sternum bones, and forelimb and hindlimb buds | ˄ Glycosphingolipid biosynthesis and glycan degradation. | ˄ Voluntary skeletal muscle contraction and twitch skeletal muscle contraction |
| **Epidermis** | Pre-epidermal keratinocytes | Epidermal cells | ˄ OXPHOS and glycolysis/gluconeogenesis.  ˅ Cholesterol metabolism. | ˄ Immune response to external stimulus.  ˅ Lipoprotein particle remodeling |
| **Multiple structures** | Fussing epithelium | Rearrangement of neighboring epithelial structures to form a unified tissue | ˄ Glycosphingolipid biosynthesis and glycan degradation. | NA |
|  | Mixed mesoderm | Cluster that contains a combination of mesoderm cell lineages | ˅ Vitamin digestion, cholesterol metabolism, fat digestion, and PPAR signaling. | ˅ Negative regulation of phospholipid biosynthetic process and vitamin transport. |
